# Lifestyle and Lipoprotein(a) Levels: Does a Specific Counseling Make Sense?

**DOI:** 10.3390/jcm13030751

**Published:** 2024-01-28

**Authors:** Federica Fogacci, Valentina Di Micoli, Pierre Sabouret, Marina Giovannini, Arrigo F. G. Cicero

**Affiliations:** 1 Hypertension and Cardiovascular Risk Research Center, Medical and Surgical Sciences Department, Alma Mater Studiorum University of Bologna, 40100 Bologna, Italy; valentina.dimicoli2@unibo.it (V.D.M.); marina.giovannini3@unibo.it (M.G.); 2 INSERM UMRS_1166, Cardiology Institute, Pitié Salpêtrière Hospital (AP-HP), ACTION Study Group, Sorbonne University, 75013 Paris, France; cardiology.sabouret@gmail.com; 3 Cardiovascular Medicine Unit, Heart, Thoracic and Vascular Department, IRCCS Azienda Ospedaliero-Universitaria di Bologna, 40100 Bologna, Italy

**Keywords:** diet, lifestyle, lipoprotein(a), nutraceuticals, physical activity

## Abstract

Lipoprotein(Lp)(a) is a variant of low-density lipoprotein (LDL), bound to apolipoprotein B100, whose levels are associated with a significant increase in the risk of atherosclerosis-related cardiovascular events, but also to aortic stenosis and atrial fibrillation. Since plasma levels of Lp(a) are commonly considered resistant to lifestyle changes, we critically reviewed the available evidence on the effect of weight loss, dietary supplements, and physical activity on this risk factor. In our review, we observed that relevant body weight loss, a relatively high intake of saturated fatty acids, the consumption of red wine, and intense physical exercise seems to be associated with significantly lower plasma Lp(a) levels. On the contrary, foods rich in trans-unsaturated fatty acids are associated with increased Lp(a) levels. With regard to dietary supplements, coenzyme Q10, L-Carnitine, and flaxseed exert a mild but significant lowering effect on plasma Lp(a).

## 1. Introduction

It is well-known that therapeutic lifestyle changes combining physical activity, diet, and weight management usually have a positive impact on metabolic risk factors and the risk of developing CV diseases [1].

Lipoprotein(Lp)(a) is a variant of low-density lipoprotein (LDL), bound to apolipoprotein B100 and characterized by an apolipoprotein(a) (apo(a)) of a different length, whose plasma concentration is usually considered unchangeable in response to lifestyle modifications [2]. During the last decades, thanks to a number of significant epidemiological investigations, high Lp(a) plasma levels have been definitely associated not only with a significant increased risk of atherosclerosis-related CV events (namely stroke, coronary artery disease, and peripheral artery disease) [3], but also with aortic stenosis and atrial fibrillation, after adjustments for other known risk factors [4,5].

According to the findings from a recently published systematic review and meta-analysis, pooling data from 75 cohort and case-cohort studies (n = 957,253), the risk of all-cause mortality increases with progressively higher levels of Lp(a), with the hazard ratios (HRs) that compare the top vs. bottom tertiles of Lp(a) being 1.18 (95% confidence interval (CI): 1.04 to 1.34) in patients in secondary prevention for CV diseases and 1.09 (95%CI: 1.01 to 1.18) in the general population. The HRs for CV diseases mortality was 1.33 (95%CI: 1.11 to 1.58) in the general population and 1.25 (95%CI: 1.10 to 1.43) in patients in secondary prevention for CV diseases. For each 50 mg/dL rise in Lp(a) plasma levels, a 31% and 15% greater risk of CV death was also estimated, respectively, in the general population and in patients in secondary prevention for CV diseases [6].

Lp(a) plasma levels are strictly genetically determined [7], so there is poor response to both lifestyle changes and currently available pharmacological drugs [8]. The prothrombotic action of Lp(a) is partially counteracted using antiplatelets, but this effect is more evident in secondary prevention patients, who should be taking antiplatelets anyway [9]. Currently, there is ongoing development of novel lipid-lowering medications, specifically small interfering RNA (siRNA) agents, such as olpasiran, SLN360, LY3819469, and the second-generation antisense oligopeptide, pelacarsen. These drugs selectively disrupt the synthesis of Lp(a) in the liver by impeding the translation of apolipoprotein(a) mRNA [10]. The primary goal is to genetically silence the lipoprotein(a) gene (LPA), diminish apolipoprotein(a) production, and subsequently, reduce serum Lp(a) levels. Current evidence indicates that optimal results are achieved through monthly subcutaneous injections, leading to persistent and substantial reductions in Lp(a) levels of up to 95%, with the potential to reduce cardiovascular risk [11]. A relevant Lp(a)-lowering effect (up to −65%, resulting in Lp(a) plasma levels of less than 50 mg/dL in 93% of participants) has been recently shown in a phase 2 trial using muvalapalin, a small oral drug [12]. The potential of these emerging drugs under development appears highly promising, demonstrating an overall safety profile. However, their cost-effectiveness will undergo thorough evaluation as an integral component of optimizing healthcare investments in CV prevention.

However, since these drugs are currently under investigation and tested only in patients already struck by coronary artery disease with very high Lp(a) plasma levels, we have no drugs in development to manage patients in primary prevention with high Lp(a) levels.

In this context, the aim of this review is to summarize the evidence supporting the ability of lifestyle interventions to improve the Lp(a) plasma levels in the context of CV management in hyperLp(a) patients.

## 2. Dietary Intervention

### 2.1. Weight Loss and Lp(a) Levels

Low-energy diets are usually associated with a global improvement in plasma lipid concentrations [13,14]. Bariatric surgery also improves plasma lipid levels [15,16,17]. However, what do we know about energy restriction and the effect of bariatric surgery on Lp(a) plasma levels? The clinical evidence of different weight loss approaches on Lp(a) plasma levels are summarized in Table 1.

The study by Kiortsis et al., involved 62 healthy obese patients (21 men aged 32 ± 9.6 years and 41 women aged 37 ± 14.6 years) consuming a low-energy diet for six months. In considering the whole population, the authors observed a body weight loss of 7.5% versus baseline and a statistically significant decrease in plasma levels of total cholesterol, LDL cholesterol, and triglycerides (*p* < 0.001), while no changes in Lp(a) levels were detected. However, when considering individuals with high Lp(a) values (>20 mg/dL) at baseline, a 17.6% reduction in Lp(a) (*p* < 0.05) was observed, which was closely related with baseline Lp(a) levels (r = 0.81 *p* < 0.001), but not with the changes in anthropometric measurements that occurred during weight loss [18]. Therefore, a low-calorie diet that induces weight loss in individuals with obesity may have positive impacts on serum Lp(a) levels, particularly in patients with elevated pretreatment concentrations of Lp(a). However, this effect is influenced by large interindividual variability and depends on the characteristics of the patients.

In the study by Berk et al. [19], after a similar 3–4 month calorie-restricted diet, patients with type 2 diabetes and obesity (n = 131) experienced a significant decrease in body weight (−9.9%), while their Lp(a) plasma levels increased by 14.8 nmol/L. Obese individuals (n = 30) or type 2 diabetics (n = 26) also experienced a decrease in body weight (−7%) associated with an increase in Lp(a) (+12.7 nmol/L), while Lp(a) did not change in obese individuals (n = 26) that underwent bariatric surgery despite considerable weight loss (−14%) [19]. The same research group carried out two further independent long-term clinical trials involving 293 overweight or obese individuals. The first study was designed as a prospective two-arm clinical investigation, including 82 patients undergoing a 7-week low-energy diet followed by Roux-en-Y gastric bypass and a 52-week follow-up (surgery group), and a control cohort of 77 patients undergoing a 59-week low-energy diet and exercise program (lifestyle group). The second study included a third cohort of 134 patients undergoing a 20-week low/very low-energy diet program (lifestyle cohort). In the lifestyle group and in the lifestyle cohort, the Lp(a) plasma level [median (interquartile range)] increased by 36% [14(7–77) vs. 19(7–94) nmol/L, *p* < 0.001] and 14% [50(14–160) vs. 57(19–208) nmol/L, *p* < 0.001], respectively. In contrast, in the surgery group, the Lp(a) levels dramatically decreased by 48% after intervention [21 (7–81) vs. 11(7–56) nmol/L, *p* < 0.001]. Remarkably, arachidonic acid and total n-3 fatty acids (FA) decreased after surgery but increased after lifestyle interventions, while plasma levels of total saturated FA remained unchanged after surgery but decreased after lifestyle interventions. However, the change in Lp(a) seemed to be independent of the weight loss [20].

In 2018, Gomez-Martin et al. [21] demonstrated that vertical sleeve gastrectomy and gastric bypass have a favorable impact on serum lipids at one-year post-surgery in women with high CV risk. This effect includes a reduction in total cholesterol, triglycerides, and oxidized-LDL, although there were no associated changes in Lp(a) levels. On the other hand, in a more recent and very large study (n = 702), Paredes et al. evaluated the 1-year metabolic impact of vertical sleeve gastrectomy. According to the findings of the study by Paredes et al., patients without metabolic syndrome (n = 372) experienced a decrease in Lp(a) levels (14.7 mg/dL vs. 12.3 mg/dL, *p* = 0.006) after vertical sleeve gastrectomy, while patients with metabolic syndrome did not (13.9 mg/dL vs. 14.6 mg/dL, *p* = 0.302). The regression model showed that older age and delta HDL-C significantly predicted the change in Lp(a), while the higher the number of metabolic syndrome components and the lower the estimated body fat percentage loss, the lower the odds of Lp(a) reduction after the intervention [22].

Even if the above cited studies are relatively small and designed differently, in particular with regard to diet composition, overall we could conclude that a low-energy diet is not sufficient to significantly modify Lp(a) levels in plasma, therefore, a more dramatic intervention is needed. The diet with a metabolic impact most like that which follows bariatric surgery is the very-low carbohydrate/ketogenic diet. In a single case of a 55-year-old triathlete with a BMI of 24.9 kg/m^2^, the use of a very-low carbohydrate/ketogenic diet was associated with a plasma Lp(a) decrease ranging from 26% to 39% in different dietary intervention phases [23]. In a large trial involving 164 overweight or obese patients (BMI: 32.4 ± 4.8 kg/m^2^) with mixed dyslipidemia, a 20-week treatment with three different weight loss diets (one of which was a low-carbohydrate diet) exerted different effects on Lp(a). These diets were comprised of 20% protein and various contents of carbohydrates and saturated fats (low-carbohydrate diet = 20% carbohydrates, 21% saturated fats; moderate-carbohydrate diet: 40% carbohydrates, 14% saturated fats; high-carbohydrate diet: 60% carbohydrates, 7% saturated fats). At the end of the study, plasma Lp(a) was reduced by nearly 15% (−14.9%; 95% confidence interval (CI): −22.0 to −7.1) only in the group randomized to the low-carbohydrate diet. The low carbohydrate diet was also associated with a significant improvement in insulin-resistance, triglycerides, HDL-cholesterol, and adiponectin plasma levels [24]. It must be acknowledged that these findings were not confirmed in a recent 12-week randomized clinical trial examining the effect of a very low-carbohydrate/high-fat diet associated with a high-intensity interval training program in a cohort of 91 overweight individuals [25]. In this study, Lp(a) plasma levels did not change, neither in response to training (n = 22), nor diet (n = 25), nor in the training + diet combined intervention (n = 25), nor standard management (n = 19) [25].

Even if these clinical trials are relatively small and short-term, their results supported the European Society of Atherosclerosis (EAS) consensus on hyper-Lp(a) management; to follow a low carbohydrate diet to reduce the concentration of Lp(a) by ~15% [26].

### 2.2. Dietary Fats

Dietary patterns abundant in animal-derived proteins and fats might be linked to increased risks of CV disease and related mortality when compared to diets rich in plant-derived protein [27,28,29]. Once again, the evidence supporting the intake of specific fatty acids and Lp(a) plasma levels is conflicting.

In overweight and obese individuals (n = 31), a 4-week plant-based diet was associated with an improvement in inflammatory and atherogenic biomarkers, among them Lp(a) (−32 nmol/L) [30]. The existing literature indicates that replacing saturated fats with an equivalent amount of unsaturated fats leads to decreased overall mortality [31]. Including nuts in the diet is a practical strategy to boost unsaturated fat intake, as it relates to lower all-cause mortality and mortality specific to CV diseases, in particular [32]. A diet abundant in walnuts, rich in alpha-linolenic acid, polyphenols, plant sterols, and tocopherol, demonstrated an overall enhancement in the blood lipid profile [33]. However, in a randomized, prospective, controlled, crossover, clinical study involving 194 healthy volunteers, an 8-week regimen of 43 g of walnuts daily did not exert any effect on Lp(a) concentration in plasma, even if other lipid fractions (such as non-HDL-C, apoB, total cholesterol, LDL-C, very LDL (VLDL) cholesterol, and triglycerides) were improved [34]. During a 16-week randomized controlled trial, 29 participants classified as overweight or obese (with a BMI of 25–40 kg/m^2^) were assigned to either consume 42.5 g/day of a mix of nuts (including cashews, almonds, macadamia nuts, Brazil nuts, pecans, pistachios, walnuts, and peanuts) or 69 g/day of isocaloric pretzels. There was no evidence that consumption of mixed nuts had an effect on LDL-C or Lp(a) throughout the intervention [35].

On the contrary, in a small study mainly enrolling Afro-Americans (n = 18/28), with a mean age of 48.3 ± 12.5 years (17 men, 11 women), Lp(a) plasma levels were negatively associated with absolute (grams/day) and relative (percentage of total calories) dietary saturated fatty acid (SFA) intake (R = −0.43, *p* = 0.02, SFA (% CAL): R = −0.38, *p* = 0.04), palmitic acid intake (R = −0.38, *p* = 0.05), and stearic acid intake (R = −0.40, *p* = 0.03) [36].

Coconut oil might also yield more favorable effects on Lp(a) concentration when compared to unsaturated oils with longer carbon chains. In a controlled crossover study involving young women and comparing two high-fat diets over a 3-week period [37], the consumption of a coconut oil-enriched diet resulted in a 17 mg/L reduction in Lp(a) levels. In contrast, the intake of highly unsaturated long-chain fatty acids led to a 25 mg/L increase in Lp(a) levels [37]. Despite being a saturated fat, it is essential to recognize that coconut oil primarily consists of medium-chain fatty acids (e.g., lauric acid), constituting 50% of its content. Consequently, it may elicit a different response in Lp(a) concentration. However, a more recent randomized, controlled, single-blinded, crossover, clinical trial that enrolled 40 healthy volunteers to investigate the short-term effect of a diet enriched in palm oil, cocoa butter, or extra virgin olive oil, with oleic acid primarily at the sn-2 position (66%, 75%, 87% sn-2 oleic acid, respectively) of the TG molecule, concluded that not one of the tested diets had a significant impact on Lp(a) levels [38].

Despite the slight benefit observed in Lp(a) levels with the consumption of coconut oil, the International guidelines advise that dietary unsaturated fats should constitute less than 10% of total energy intake [39,40]. This recommendation stems from the association of excess unsaturated fat consumption with significantly elevated morbidity and mortality rates related to cancers and CV diseases [27,31].

Trans-fatty acids are associated with adverse CV outcomes. Whether a part of this effect is mediated by an impact of trans-fatty acids on Lp(a) levels is not clear [41].

In a double-blind clinical trial involving 29 men and 29 women, the participants were randomized to eat one of four controlled diets for six weeks each, where fatty acids accounted for 39 to 40% of energy: (A) oleic (16.7% of energy as oleic acid); (B) moderate trans (3.8% of energy as trans monoenes, approximately the trans content of the U.S. diet); (C) high trans (6.6% of energy as trans monoenes); (D) saturated (16.2% of energy as lauric, myristic, and palmitic acids). The saturated diet significantly reduced Lp(a) levels from 8% to 11%. Compared with the oleic diet (A), the trans diet had no adverse effect on Lp(a) levels in the whole cohort. However, the subgroup of individuals with higher Lp(a) levels at baseline (≥30 mg/dL) responded to the high trans diet (C) with a slight, though significant increase (+5%) in Lp(a) levels compared to the oleic (A) and moderate trans (B) diets [42].

In a small, randomized, clinical trial involving 31 young men, the consumption of hydrogenated soybean oil led to a notably higher level of Lp(a) compared to a diet primarily sourced from butter [43]. On the contrary, in a randomized, crossover study with 49 hypercholesterolemic patients following a 6-week diet rich in either butter or margarine, there was no observed change in Lp(a) [44]. Intake of meals high in specific dietary fatty acids can increase postprandial plasma lipids differently [45,46,47,48], including Lp(a) concentration. In a clinical trial enrolling healthy, young men, 16 volunteers were asked to sequentially consume five test fats dominated by (approximately 43% g/kg) stearic, palmitic, oleic, C18:1 trans, or linoleic acid incorporated into meals (1 g fat/kg body weight) after a 12-h fast, in random order on different days, separated by 3-week washout periods. Blood samples were drawn before, and 2, 4, 6, and 8 h after eating. Lp(a) plasma levels were found to increase after each supplementation, except after oleic and C18:1 trans consumption. On the contrary, oleic and C18:1 trans supplementation was associated with less area under the plasma Lp(a) concentration curve compared to those measured after stearic and palmitic acid intake (*p* < 0.003). So, long-chain stearic and palmitic acids led to significant increases in postprandial Lp(a) levels after an oral fat test in young, healthy men [49].

The clinical evidence of different dietary fatty acids on Lp(a) plasma levels have been summarized in Table 2.

In conclusion, based on the available data, mainly obtained in small and short-term clinical trials, a mild increase in plant-derived saturated fatty acids could mildly decrease the Lp(a) plasma levels, while trans-fatty acid rich foods should be avoided. The available evidence could not be translated to a suggestion to increase saturated fatty acids in a diet aiming to improve LDL-C and reduce CV risk, however, it suggests that in individuals with high Lp(a) levels, an extreme reduction of saturated fatty acids is not mandatory and probably also negative.

### 2.3. Popular Beverages

A variable consumption of beverages such as coffee, tea, and alcohol worldwide may be correlated with CV outcomes [50,51,52]. Thus, the potential effects of popular beverages on Lp(a) concentration deserves consideration.

In 15 mildly hypercholesterolemic adults (mean LDL-C = 135 mg/dL) consuming five cups/day of black tea prepared using 180 mL of water for each serving, Lp(a) decreased by 16% as compared with placebo [53]. Of course, the number of individuals enrolled in this study was too small to be conclusive with regard to the potential use of black tea as a Lp(a) lowering tool and larger long-term studies should be designed to confirm or disprove this observation. In another study, 53 volunteers with diabetes were randomly assigned to drink either black tea (n = 26) or *Hibiscus sabdarrifa* tea (n = 27), by using 2 g of tea sachet with 240 mL of boiling water for each serving, twice daily for 1 month. The Lp(a) concentrations remained unchanged from the baseline value of 26 mg/dL in both study groups [54]. Further research is needed, especially with respect to the number of commercially available tea preparations.

A rapidly increasing body of evidence recognizes the potential benefits of coffee in relation to CVD [55,56,57,58], but its effects on Lp(a) plasma levels remain unclear. Moreover, the exact mechanism by which coffee or single coffee components affect Lp(a) levels is yet to be clarified. The type of coffee and method of preparation appear to be important in determining the effect on Lp(a); in fact, coffee diterpenes present in unfiltered coffee brews are among the few dietary constituents that may modulate Lp(a) levels [59]. According to the findings of a systematic review and meta-analysis, the consumption of coffee or coffee diterpenes was associated with either a reduction in Lp(a) of 11 mg/dL (6 trials, 275 individuals), or no effect (2 trials, 56 individuals) [60]. However, it must be recognized that this metanalysis was affected by a large inter-study heterogeneity as regards study design, type of intervention, coffee source, and method of coffee processing [60].

A cross-sectional study with 309 volunteers showed that serum Lp(a) was elevated in chronic boiled coffee drinkers, who had a median Lp(a) of 13.0 mg/dL (range 0–130) compared with filter coffee drinkers who had a median Lp(a) of 7.9 mg/dL (range 0–144). The effect of coffee on Lp(a) is complex and may follow a biphasic time course, that is to say that whilst coffee may have a short-term beneficial effect in reducing Lp(a), in the longer term it may prove to be detrimental [61]. On the other hand, in the large UK Biobank database (n = 447,794 participants aged 37–73 years) no association was observed between coffee or tea intake and Lp(a) plasma levels [62].

In a cross-sectional study involving 300 middle-aged men, the Lp(a) concentrations in subgroups with low (<39 g/week), intermediate (39–132 g/week), and high (>132 g/week) ethanol intake were 137, 109, and 94 mg/L, respectively (P between groups < 0.05). Interestingly, abstainers exhibited a higher Lp(a) concentration (median, 206 mg/L) compared to drinkers [63]. However, in another cross-sectional study of 402 subjects with untreated hypertension, those with light (1–20 g/d), moderate (20–50 g/d), and heavy (>50 g/d) ethanol consumption showed 21%, 26%, and 57% lower median Lp(a) concentrations, respectively, compared to abstainers and occasional drinkers [64]. Notably, red wine consumption appears to have a greater ability to decrease Lp(a) levels than white wine. In a study involving 20 healthy male volunteers, the daily intake of 200 mL of red wine for 10 days resulted in a reduction in Lp(a) levels from 18.6 to 13.2 mg/dL (*p* < 0.001), whereas a similar effect was not observed with white wine after a 6-week washout period [65]. Of course, the study was too small and short-term to furnish strong evidence that red wine more effectively reduces Lp(a) plasma levels than white wine. In a 4-week randomized crossover study in 67 men with high estimated CV risk, Lp(a) levels were compared after the ingestion of red wine (30 g alcohol/day), the equivalent amount of dealcoholized red wine, and gin (30 g alcohol/day) [66]. The Lp(a) level fell from 54.4 mg/dL (baseline value) to 50.2 mg/dL, only after the intervention with red wine [66]. The conflicting results of the different studies suggest the need of more in depth research on larger cohorts, focusing on the different kinds of alcohol consumed (beer, red wine, white wine, shots of spirits). In any case, the adverse health effects of more than minimal alcohol intake may well outweigh any potential benefit in lowering Lp(a) levels [67,68].

## 3. Nutraceutical Supplementation

Several lipid-lowering nutraceuticals and functional foods have shown to significantly reduce the plasma levels of LDL-cholesterol, however, most of them have no effect on Lp(a) [69]. L-carnitine and coenzyme Q10 [70,71], are the nutraceuticals most studied for Lp(a) lowering effects, followed by flaxseed and curcumin. Of course, the available trials are small and short-term but somewhat suggestive of the positive effects of these dietary supplements.

### 3.1. L-Carnitine

Levo-Carnitine (L-carnitine) is an amino acid present in a number of foods, especially in meat [70]. Both oral and intravenous L-carnitine administration may provide benefits in individuals affected by CV disease [72,73,74,75], mainly because of its antioxidant and energy metabolism improvement actions [73,76]. However, the amount of L-carnitine provided by foods is much lower than that needed to achieve a reduction in Lp(a) in humans (Table 3).

A meta-analysis of data derived from seven double-blind, randomized, clinical trials (n = 300) showed a significant reduction in Lp(a) levels following L-carnitine supplementation (weighted mean difference (WMD): −8.82 mg/dL, 95% CI: −10.09, −7.55, *p* < 0.001) for 1–24 weeks. When studies were classified according to the route of administration, a significant reduction in plasma Lp(a) concentration was observed with L-carnitine by the oral route (WMD: −9.00 mg/dL, 95% CI: −10.29, −7.72, *p* < 0.001), but not by the intravenous route (WMD: −2.91 mg/dL, 95% CI: −10.22, 4.41, *p* = 0.436). The results of the meta-regression analysis showed that the pooled estimate was independent of L-carnitine dose (slope: −0.30; 95% CI: −4.19, 3.59; *p* = 0.878) and duration of therapy (slope: 0.18; 95% CI: −0.22, 0.59; *p* = 0.374) [70].

This effect was confirmed by a more recent meta-analysis evaluating the metabolic effect of L-carnitine supplementation, concluding that L-carnitine is able to reduce Lp(a) levels by a mean value of 7.13 mg/dL [95% CI: −9.82, −4.43] mg/dL; *p* < 0.001) [76].

Additionally, in individuals with mixed hyperlipidemia, Florentin et al. [77] demonstrated that the coadministration of 2 g/day of L-carnitine with 20 mg/day of simvastatin over a 12-week period resulted in a reduction of Lp(a) levels from 56 to 42 mg/dL. This benefit was not observed with simvastatin monotherapy, suggesting that the decrease in Lp(a) could be attributed to the presence of L-carnitine. The middle-term tolerability and safety of L-carnitine is high. Therefore, longitudinal studies are needed in order to provide information about whether potential benefits of L-carnitine on Lp(a) level outweigh or modulate the atherosclerotic and metabolic damage associated with increased plasma levels of trimethylamine N-oxide. In fact, L-carnitine supplementation may unfortunately raise trimethylamine N-oxide production in the liver [78,79,80] and thus, trimethylamine N-oxide plasma levels, which is a well-known CV risk factor [81].

### 3.2. Coenzyme Q10

Coenzyme Q10 (CoQ10) is a powerful antioxidant, plays an essential role in the respiratory chain as an electron carrier in mitochondrial ATP synthesis [82,83], and exerts an anti-inflammatory action [84]. Similarly to L-carnitine, coenzyme Q10 (CoQ10) supple-mentation may benefit individuals with CV diseases [85,86], but with dosages that cannot be reached by dietary intake (Table 4).

A meta-analysis of seven double-blind, randomized, clinical trials found that CoQ10 supplementation was accompanied by a slight but significant reduction in plasma Lp(a) levels (WMD: −3.54 mg/dL, 95% CI: −5.50, −1.58; *p* < 0.001), an effect more robust in studies with higher baseline Lp(a) levels (slope: −0.44; 95% CI: −0.80, −0.08; *p* = 0.018). The reduction in plasma Lp(a) levels was consistent with different doses of CoQ10, with an inverse association between the dose of CoQ10 administered and the reduction in Lp(a) (slope: 0.04; 95% CI: 0.01, 0.07; *p* = 0.004) [87]. The positive effect of CoQ10 supplementation on Lp(a) plasma levels was not confirmed in a later, but smaller meta-analysis focusing only on patients affected by coronary artery disease [88], or in a further double-blind, randomized, clinical trial more recently carried out in 60 type 2 diabetics [89].

CoQ10 supplementation is tolerable and safe. Its efficacy as a Lp(a) lowering agent is mild and the identification of its most cost-effective daily dose deserves further research.

### 3.3. Flaxseeds

Flaxseed (*Linum usitatissimum* L.) is a rich source of alpha-linolenic acid, whose supplementation has been demonstrated to mildly, but significantly decrease plasma Lp(a) levels (standardized mean difference: −0.22, 95% CI: −0.41 to −0.04, *p* = 0.017) in a meta-analysis of six double-blind, randomized, placebo-controlled clinical trials [89].

The effect is small, the trials are few and mainly short-term, so that further evidence is needed to support supplementation with flaxseed in hyperLp(a) patients.

### 3.4. Curcumin

Some clinical trials suggest a positive impact of turmeric extracts on Lp(a) levels.

The first evidence came from a double blind, placebo-controlled clinical trial carried out on 100 Iranian patients affected by metabolic syndrome and randomized to assume curcuminoids (1000 mg/day plus piperine 10 mg/day; n = 50) or placebo (n = 50) for eight weeks [90], with between-group changes of 4.55 mg/dL (−5.00 to 0.00, *p* < 0.001). The same research group confirmed the results in a cohort of 118 type 2 diabetes patients randomized to be treated with curcuminoids (1000 mg/day plus piperine 10 mg/day) or placebo on top of standard care. Beyond a global positive impact on the plasma lipid patterns, Lp(a) decreased by −1.5 ± 1.6 in the active treatment group versus −0.3 ± 1.7 in the placebo treated group (*p* = 0.001) [91].

Finally, these results have been recently confirmed in a double-blind, placebo-controlled clinical trial on type 2 diabetic patients (n = 64), and mild to moderate coronary artery disease (<70% stenosis in angiography), randomized to receive nanosomal-curcumin (80 mg/day) or placebo on top of optimal medications for 90 days [92].

## 4. Physical Activity

It is well-known that physical activity is associated with a reduced risk of CV disease [93]. Since regular exercise is associated with favorable changes in blood lipoproteins, in particular to an increase in HDL-cholesterolemia and a decrease in triglyceridemia, the question was raised whether there might be any correlation between serum Lp(a) levels and physical activity [94]. Population and cross-sectional studies usually show a lack of association between serum Lp(a) levels and regular and moderate physical activity [95].

An exception is a large Finnish study carried out on children and young adults aged 9, 12, 15, 18, 21, and 24 years (n = 2464, where the Lp(a) ranged from <2 to 90.8 mg/dL) [96]. A physical activity index was specifically calculated for this study, where the serum Lp(a) concentration was significantly correlated with the physical activity level, independently from age and gender, and elevated Lp(a) levels (>25 mg/dL) were less frequent in more physically active subjects.

In a further small cross-sectional study carried out on 80 young patients affected by type 1 diabetes, physical activity was assessed using pedometers measuring the total number of steps per week. Here, a habitual intermediated intensity physical activity was associated with lower Lp(a) plasma levels [97].

However, some cross-sectional studies suggest that serum Lp(a) levels increase in response to intense load training (2–3 h per day), such as distance running or weight-lifting, over several months or years. These changes usually range between a 10 and 15 percent increase [98]. It is unclear whether increased serum Lp(a) levels after intense training or whether physical activity associated with favorable Lp(a) levels have clinical relevance, or whether possibly some isoforms of Lp(a) are more sensitive to the effects of training [98].

Based on the available evidence, it is hard to draw any conclusion on the relationship between Lp(a) plasma levels and different kinds of physical activities or training, because of the large heterogeneity of the available studies, the different methodologies used to record or estimate physical activity, the lack of data on different ethnicities, and on the measure of the different apo(a) isoforms.

Overall, there is inconclusive evidence that standard physical activity improves Lp(a) plasma concentrations, but some intensive training could be suggested in patients able to afford it. However, this evidence also is mainly based on the results of small and short-term trials.

## 5. Discussion

Hyperlipoproteinemia(a) is largely prevalent in the general population [99]. Statins have no effect on Lp(a) plasma levels, even if partly balancing the negative effect of Lp(a) on CV risk when plasma Lp(a) concentrations are less than 50 mg/dL [100]. Slow-release nicotinic acid is the only drug able to reduce Lp(a) by 20–30%, but it is not well-tolerated and its long-term safety has been questioned [101]. Mipomersen, an anti-sense oligonucleotide directed against apolipoprotein-B 100 mRNA in the liver, is also able to reduce Lp(a) levels, but its liver safety has been seriously questioned as well [102]. The cholesteryl ester transfer protein (CETP) inhibitors increase the HDL fraction while decreasing the atherogenic non-HDL particles, such as Lp(a) [103]. A meta-analysis of ten randomized clinical studies (34,781 patients overall) found that anacetrapib significantly lowers plasma Lp(a) level by a weighted mean difference of −13.35 (95%CI: −18.31 to −8.39) [104]. Another CETP inhibitor, evacetrapib has been shown to reduce Lp(a) by 30–40% over a period of 12 weeks [105]. However, once again, not one of these drugs has been associated with a CVD risk reduction.

In cohort studies, plasma proprotein convertase subtilisin/kexin type 9 (PCSK9) relates with Lp(a) plasma levels [106]. On the other hand, the PCSK9 inhibitors have been shown to significantly, though mildly, reduce Lp(a) plasma levels [107,108]. A recent network meta-analysis of 41 randomized controlled trials with 17,601 participants concluded that the available PCSK9 inhibitors are able to significantly reduce Lp(a) plasma levels (by up to 25.1%), more evidently with Evolocumab and Alirocumab than with Inclisiran [109,110]. However, they are expensive and their use is limited to patients with high-to-very high CV risk.

In our review, we observed that relevant body weight loss, a relatively high intake of saturated fatty acids, the consumption of red wine, and intense physical exercise seems to be associated with significantly lower plasma Lp(a) levels. On the contrary, foods rich in trans-unsaturated fatty acids are associated with increased Lp(a) levels. As regards dietary supplements, coenzyme Q10, L-Carnitine, and flaxseed exert a mild but significant lowering effect on plasma Lp(a).

Of course, other bioactive compounds have been supposed to exert a Lp(a) lowering effect, but their efficacy is even more questionable. For instance, different experimental models suggest that Vitamin C can modulate Lp(a) synthesis [111]. However, two randomized clinical trials did not show any Lp(a)-reducing effect of Vitamin C supplementation. The first one tested the effect of eight months of supplementation with 1 g/day vitamin C in healthy subjects [112], while the second one investigated the effect of 12 weeks of supplementation of 4.5 g/day vitamin C in patients affected by premature CV disease [113]. On the other hand, vitamin B3 (nicotinic acid) exerts a significant Lp(a) lowering effect, but at pharmacological dosages, i.e., at least 30 times the maximum daily dosage recommended as a dietary supplement (35 mg), that are also usually associated with disturbing side effects [114].

Beyond a small study carried out on 40 patients treated with peritoneal hemodialysis, in which 100 mg of soy isoflavones were associated to a significant reduction in Lp(a) levels (up to 10%) after eight weeks of intake [115], a meta-analysis of ten trials involving 973 subjects concluded that isoflavone supplementation had no effect on reducing Lp(a) levels [116].

Dietary factors other than those considered in this review could be associated with Lp(a) plasma levels. For instance, a recent cross-sectional study carried out on 7662 US adults showed a positive correlation between plasma Lp(a) and serum carotenoids (lycopene, lutein, beta-cryptoxanthin), beta-carotene, and alfa-carotene, but a negative association with serum vitamin B12 and folate, whose clinical significance has yet to be clarified [117].

Overall, a healthy diet is suggested for patients with high Lp(a) levels. Body weight reduction seems to be advisable. The most cost-effective way to quickly achieve the optimal body weight in these patients seems to be the ketogenic diet, but its chronic use should not be recommended [118], because of its possible negative affect on LDL-cholesterolemia. The dietary pattern change should be associated with intensification of physical activity. Among the potentially useful and safe dietary supplements, coenzyme Q10 and flaxseed could be considered. It could be argued that different life-style approaches should be considered for the management of high Lp(a) plasma levels in patients with pure hyperLp(a) and in those with high Lp(a) associated with hypercholesterolemia or mixed hyperlipidemia (Table 5). Of course, this is only a speculative suggestion. In fact, for the most part, currently available evidence supporting specific life-style interventions to reduce plasma Lp(a) levels come from cross-sectional studies and small intervention trials. There is a total lack of evidence that Lp(a)-induced changes by dietary and behavioral habits are also related to specific CV outcomes.

We also acknowledge some main limitations of this review. First, a relevant part of the available literature is relatively old. This is probably since Lp(a) has been classified as a non-modifiable risk factor, which has slowed the research on the factors that are possibly related to its modification until recent years. Second, the available evidence is related to a few epidemiological trials and to small short-term clinical trials, whose methodology is not always of high quality.

## 6. Conclusions

A low-energy, relatively high in saturated fatty acid diet, intense physical activity, and some dietary supplements have a mild but significant effect on plasma Lp(a) levels. Further studies are needed to confirm the available evidence and to test an Lp(a) reduction lifestyle and its impact on the risk of developing CV diseases.

## Figures and Tables

**Table 1:** Clinical evidence of different weight loss approaches on Lp(a) plasma levels.

First Author, Year of Publication	Patients	Interventions	Main Result
Kiortsis et al., 2001 [18]	Healthy obese (n = 62)	6-week low-energy diet	17.6% Lp(a) reduction in subjects with Lp(a) > 20 mg/dL only
Berk et al., 2017 [19]	Obese and Type 2 diabetes (n = 131) Obese (n = 30) Type 2 diabetes (n = 26) Obese managed with bariatric surgery (n = 26)	4-month low-energy diet or bariatric surgery	Lp(a) increase in subjects undergoing low-energy diet, 14% Lp(a) decrease in subjects treated with bariatric surgery
Berk et al., 2022 [20]	Overweight/Obese patients (n = 293)	7-week low-energy diet followed by Roux-en-Y gastric bypass) and 52-week follow-up (surgery group) (n = 82) 59-week low-energy diet and exercise program (lifestyle group) (n = 77) 20-week low/very low energy diet (lifestyle cohort) (n = 134)	Lp(a) increase in after low/very low energy diet, 48% Lp(a) decrease after surgery
Gomez-Martin et al., 2018 [21]	40 obese women	1-year follow-up after laparoscopic Roux-en-Y gastric bypass (n = 20) or sleeve gastrectomy (n = 20) or conventional treatment with diet and exercise (n = 20)	No change in Lp(a) plasma level in any group
Paredes et al., 2020 [22]	702 obese patients (372 without metabolic syndrome)	1-year follow-up after vertical sleeve gastrectomy	10% decrease in Lp(a) levels in metabolic syndrome only
Scholl et al., 2020 [23]	Single case report of a normoweight subject	Very low carb ketogenic diet during physical training	26–39% decrease in Lp(a) levels
Ebbeling et al., 2022 [24]	164 overweight/obese subjects with mixed dyslipidaemia	20-week weight loss diet containing 20% proteins plus different amounts of carbohydrates and saturated fatty acids (20–21% vs. 40–14% vs. 60–7%)	15% decrease in Lp(a) levels in the low-carbohydrate diet group only
Cipryan et al., 2022 [25]	91 overweight subjects	High-intensity interval training program (n = 22) vs. high-intensity interval training program and very low-carb/high fat diet (n = 25) vs. very low carb/high fat (n = 22) vs. standard management	No effect of any treatment on Lp(a) plasma level)

**Table 2:** Clinical evidence of different dietary fatty acids on Lp(a) plasma levels.

First Author, Year of Publication	Patients	Interventions	Main Result
Najjar et al., 2018 [30]	31 Overweight/obese with LDL-C > 100 mg/dL	4-week plant-based diet	16% Lp(a) plasma level reduction
Bamberger et al., 2017 [34]	194 healthy subjects	8-week regimen of 43 g of walnuts daily vs. standard diet	No change in Lp(a) plasma level in any group
Nora et al., 2023 [35]	29 overweight or obese individuals	16-week consumption of either 42.5 g/day of mixed nuts (cashews, almonds, macadamia nuts, Brazil nuts, pecans, pistachios, walnuts, and peanuts) or 69 g/day isocaloric pretzels	No change in Lp(a) plasma level in any group
Mueller et al., 2003 [37]	25 healthy women	3-week coconut oil-based high fat diet vs. coconut oil-based low-fat diet vs. diet rich in mono- and polyunsaturated fatty acids	Lp(a) reduced by 17 mg/L after coconut-oil intake, but increased by 25 mg/dL after intake of unsaturated long-chain fatty acids
Loganathan et al., 2022 [38]	40 healthy women	4-week comparison of the effect of palm oil, cocoa butter, extra virgin olive oil as the main oil	No change in Lp(a) plasma level in any group
Clevidence et al., 1997 [42]	58 healthy subjects	6-week comparison of the effect of high-fat diets (fatty acids = 39–40% of total energy) characterized by oleic (16.7% of energy); trans (3.8% of energy); high trans (6.6% of energy as trans-monoenes); saturated (16.2% of energy)	8–11% decrease in Lp(a) plasma levels with high saturated fatty acids diets; 5% Lp(a) levels in subjects with higher Lp(a) at the baseline with the high trans-diet
Almendingen et al., 1995 [43]	31 young men	3-week effect of partially hydrogenated fish oil, partially hydrogenated soybean oil, and butterfat	Lp(a) plasma level increase with all diets, but larger with hydrogenated fats-enriched diets.
Chisholm, et al., 1996 [44]	49 hypercholesterolemic subjects	6-week effect of butter or an unsaturated margarine used for cooking or spreading in a reduced fat diet	No change in Lp(a) plasma level in any group
Tholstrup et al., 2004 [49]	16 young healthy men	5 test fats dominated by (approximately 43% g/kg) stearic, palmitic, oleic, C18:1 trans, or linoleic acid incorporated into meals (1 g fat/kg body weight) after a 12-h fast in random order on different days, separated by 3-week washout periods	Lp(a) plasma levels increased after each fat except oleic and C18:1 trans; oleic, C18:1 trans intake was associated with less area under the plasma Lp(a) concentration curve

**Table 3:** L-Carnitine contents in foods (modified from [70]).

Food Item	L-Carnitine (mg/100 gr Serving)	Food Item	L-Carnitine (mg/100 gr Serving)
Meat products		Dairy products	
Beef steak	64.6–87.5	Yogurt, regular (3.2% fat)	12.5
Pork (muscle)	13–53.5	Milk 2–4% fat	2.3–2.9
Chicken	10–10.4	Cheese	1.4–1.8
		Butter	0.85
Fish		Chicken egg	
Salmon (cooked)	5.8	Whole	Not evaluated
Cod (Atlantic)	1.8	Egg Yolk	0.8
		Egg white	0.3

**Table 4:** Coenzyme Q10 contents in foods (modified from [82]).

Food Item	CoQ10 (mg/100 gr Serving)	Food Item	CoQ10 (mg/100 gr Serving)
Meat products		Dairy products	
Beef steak	1.61–3.65	Yogurt, regular (3.2% fat)	0.07–0.11
Pork (muscle)	2.43–4.11	Milk 2–4% fat	0.07–0.12
Chicken	1.4–2.1	Cheese	0.12–0.13
		Butter	0.71
Fish		Chicken egg	
Salmon (cooked)	0.43–0.76	Whole	0.07–0.37
Cod (Atlantic)	0.37	Egg Yolk	Not evaluated
		Egg white	0.52

**Table 5:** Lifestyle suggestions for patients with pure isolated hyperLp(a), high plasma Lp(a) associated with hypercholesterolemia, and high Lp(a) associated with mixed hyperlipidemia.

	High Lp(a) Only	High Lp(a) + High LDL	High Lp(a) + High LDL + High TG
Cigarette smoking stop	↓↓	↓↓	↓↓
Physical activity	Intense	Moderate	Moderate-to-Intense
Body weight	↓↓	↓	↓↓
Alcohol intake	↑	↓	↓↓
Saturated fatty acids	↑	↓	↓
Unsaturated fatty acids	-	↑	↑
Trans fatty acids	↓↓	↓↓	↓↓
Ultraprocessed foods	↓↓	↓↓	↓↓
Whole foods	↑	↑	↑
Vegetables	↑	↑	↑
Dietary supplements	Coenzyme Q10, flaxseed, curcumin		

↓↓ = Clinically significant reduction; ↓ = Mild reduction, ↑ = Mild increase.

## Data Availability

Not applicable.
